# Spatiotemporal patterns of soil moisture in Shandong Province, China: An analysis using ERA5-Land datasets

**DOI:** 10.1371/journal.pone.0339023

**Published:** 2026-02-05

**Authors:** Ziqian Wang, Bo Hu, Yanxia Hu, Xianmin Wu, Luxia Liu

**Affiliations:** 1 School of Water Conservancy and Environment, University of Jinan, Jinan, China; 2 Qingdao Water Conservancy Survey and Design Institute, Qingdao, China; Swedish Meteorological and Hydrological Institute, SWEDEN

## Abstract

Understanding the spatiotemporal variations of soil moisture (SM) is crucial for the timely identification of drought and the advancement of regional water resource management. This study utilized ERA5-Land reanalysis data and employed the Mann-Kendall test, correlation analysis, wavelet transform, and Generalized Additive Models (GAM) to analyze the spatiotemporal patterns of SM from 1951 to 2024 in Shandong Province, China. The study also examined the factors influencing these patterns. The results showed that: 1.) The mean annual soil moisture exhibited a significant decreasing trend at a rate of −0.00055 mm/year, and a significant seasonal decreasing trend has been detected. Precipitation emerged as the dominant driver of soil moisture spatiotemporal variability. 2.) Spatially, soil moisture exhibited a distribution pattern consistent with the spatial configurations of precipitation and evaporation. Relationships between soil moisture and climatic variables varied significantly among regions and seasons. 3.) Wavelet analysis revealed 8-year, 16-year, and 32-year periodicities in soil moisture that aligned with the dominant oscillations observed in precipitation and evaporation. 4.) Time-lagged cross-correlation analysis revealed that soil moisture exhibited the strongest instantaneous correlation with precipitation, while this response delay became more pronounced in deeper soil layers. These findings not only provide a basis for enhanced drought monitoring and region-specific management, but also inform the development of sustainable water resource policies aligned with identified multi-year cycles.

## 1. Introduction

Soil moisture is a key component of the terrestrial hydrological cycle and a crucial factor affecting the response of runoff to precipitation [1,2]. On the one hand, soil moisture controls hydrological, meteorological, and ecological processes by distributing precipitation into infiltration, runoff, and surface storage [3]. On the other hand, it affects surface temperature and the occurrence of extreme precipitation events by partitioning effective solar radiation into sensible heat for warming and latent heat for evapotranspiration [4]. Moreover, surface soil moisture determines evapotranspiration and energy flux exchange, and directly affects the eco-hydrological processes in the soil-vegetation-atmosphere system [5]. Therefore, monitoring the spatiotemporal variations of soil moisture is of great significance for agricultural production, ecological environment, water resource management [6], and supporting hydro-meteorological research.

Currently, commonly used methods for soil moisture monitoring include in-situ station observations [7], remote sensing inversion, and model derivation [8]. The in-situ observation stations can capture real variations in soil moisture over several consecutive years with high temporal resolution (e.g., on the scale of seconds or minutes) [9]. However, due to the high cost of measurements, deploying a high-density observation network globally is challenging [10,11]. Remote sensing monitoring offers wide coverage, enabling the acquisition of real-time, large-scale, and long-term data [12,13]. However, its inversion accuracy is affected by factors such as surface cover type, terrain, and climate, and its temporal resolution is generally low due to the satellite revisit cycle. In addition, passive microwave and optical remote sensing typically only detect surface soil moisture. In contrast, the model dataset is generated by assimilating various types of data (remote sensing data, station observations, and climate data) through the model, which reduces the uncertainty in soil moisture estimates caused by differences in land cover types and climate types [14]. It can be used to infer root layer (> 5 cm) soil moisture content. Moreover, optimizing the assimilation model algorithm allows dynamic adjustments to model parameters [15], thereby improving the accuracy and spatiotemporal resolution of soil moisture monitoring. Many assessment studies have shown that most model datasets accurately capture the spatiotemporal variations of observed soil moisture data [16–18]. ERA5-Land, ERA5, FLDAS and GLDAS are reanalysis products characterized by long time series, high temporal resolution, rich parameters (including not only soil moisture from the surface to the root zone but also variables such as evaporation, runoff, and snow water equivalent), and free accessibility. These datasets can effectively capture the dynamic changes in soil moisture [19,20].

In recent years, extensive research has been conducted on the spatiotemporal variations of soil moisture across different regions. Such studies enhance the understanding of land-atmosphere interactions and climate change, enhance the precise management of agriculture and water resources, and provide decision-making support for addressing global changes. Zhou et al. [21] measured soil volumetric water content at different depths over a 10-months period using the SWR-100 probe to study vegetation’s impact on spatiotemporal soil moisture distribution. Yao et al. [22] and Wang et al. [23] employed the ESA CCI dataset to investigate soil moisture dynamics in agricultural regions of northern China and the South-to-North Water Diversion Project area in Jiangsu Province. Their studies revealed distinct seasonal variations across all study areas, with a notable aridification trend observed in northern agricultural zones, while the water diversion area in Jiangsu exhibited an overall increasing trend in soil moisture. Wang et al. [24] utilized GIMMS NDVI data from 1982 to 2015 and ERA5 soil moisture data to analyze the spatial distribution and trends of soil moisture and vegetation on the Tibetan Plateau. Geng et al. [25] used the GLDAS-Noah v2.0 and v2.1 soil layer moisture datasets to analyze the spatiotemporal distribution of soil moisture in China at depths of 0–200 cm from 1948 to 2018. Hu et al. [26] used GLDAS assimilation data to investigate the spatiotemporal variations and variability of soil moisture on an annual scale in the semi-arid region of Xinjiang, China, and explained the spatiotemporal patterns of soil moisture variations in typical semi-arid regions of Central Asia.

While studying the spatiotemporal variation characteristics of soil moisture, scholars have also conducted extensive research on the driving factors of soil moisture changes. They have found that the factors affecting soil moisture variations include natural elements (e.g., meteorological conditions, topography, soil properties, vegetation types and density, groundwater depth) [27–30] and human activity factors (e.g., land use) [31]. Li et al. [4] used satellite-based SM products and employed a GAM model to quantify both the individual and combined effects of precipitation, evapotranspiration, and vapor pressure deficit on soil moisture dynamics. Wei et al. [31] quantitatively analyzed the contributions of climate change and human activities to the temporal trends of soil moisture in the Southwest Karst region, using statistical methods (linear regression, Mann-Kendall test, and residual analysis). They found that human activities and climate change accounted for 59% and 41% of the drying trend of soil moisture, respectively. Su et al. [32] conducted a statistical analysis of soil moisture and meteorological data (1988–2013) in the Tarim River Basin, revealing precipitation as the dominant control factor, with temperature showing weaker influence. Li et al. [33] further established that elevation serves as a key determinant of spatiotemporal patterns in soil moisture distribution.

Nowadays, spatiotemporal variation patterns of soil moisture differ across regions, and their influencing factors also vary slightly [34]. Although the availability of soil moisture datasets has increased at local, regional, and even global scales, significant spatiotemporal variations in soil moisture may still exist across these scales [33]. Therefore, exploring the spatiotemporal patterns of soil moisture and their driving factors at the regional scale remains highly significant [35], providing more detailed and targeted information on the variation patterns of soil moisture in the region. As an important agricultural province in China, the soil moisture status in Shandong Province is directly related to regional food security and ecological security, so research on soil moisture in Shandong Province becomes particularly important. However, there have been few studies on the spatiotemporal variations of soil moisture over a long time series and the related driving factors in Shandong Province by previous researchers. Therefore, this study aims to reveal the spatiotemporal variation characteristics of surface soil moisture in Shandong Province from 1951 to 2024 based on the ERA5-Land reanalysis data, and mainly explore its relationship with climatic factors such as precipitation, temperature, and evaporation. This is conducive to further understanding the variation patterns and influencing mechanisms of soil moisture in Shandong Province, and can provide a scientific basis for regional water resource management and sustainable agricultural development.

## 2. Materials and methods

### 2.1. Study area

**Fig 1:**
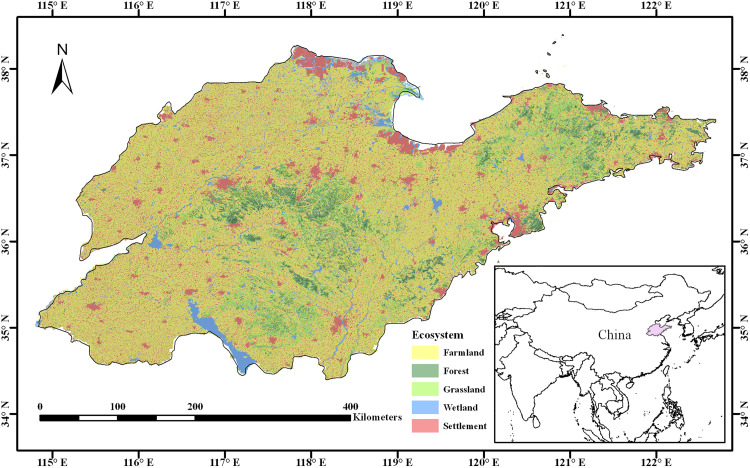
Overview of the study area. Land borders have been created using data from https://www.naturalearthdata.com/.

Shandong Province (Fig 1) is located in the eastern part of China. The topography of the province is complex, with mountainous outcrops in the center, low-lying and flat areas in the west, and gently rolling hills in the east. The climate of the region is a warm temperate monsoon climate with an average annual temperature of 11–14°C and an annual precipitation of 550–950 mm, with obvious seasonal and regional differences.

As an important agricultural province in China, Shandong Province has a large number of farmlands concentrated in its plain areas. However, due to the uneven spatiotemporal distribution of precipitation and other reasons, this region is prone to drought. The spatiotemporal variation of soil moisture has an extremely crucial influence on crop growth and agricultural yield. So, studying the spatiotemporal variation patterns of soil moisture in Shandong Province is conducive to formulating reasonable drought-prevention measures in advance and mitigating the adverse effects of drought on agricultural production.

### 2.2. Dataset

The SM data used in this study were from the ERA5-Land reanalysis dataset published by the European Centre for Medium-Range Weather Forecasts (ECMWF), covering the period from February 1950 to December 2024. This dataset was generated by combining observational data (e.g., satellites, ground stations) with numerical models, ensuring high accuracy and consistency. Existing studies have shown that the ERA5-Land SM data performs better on long term scales, and its overall performance is superior to that of satellite retrievals [36]. Moreover, it had a relatively high Pearson correlation coefficient (R) and a relatively low unbiased root mean square error (ubRMSE) with in-situ observational data [37].

This study obtained in-situ measurement data from 27 stations across China, totaling 12,473 rows, from the Chinese Ecosystem Research Network (CERN). The time span of the data ranged from January 2005 to around September 2020. On national-scale evaluation, a comparative analysis between the ERA5-Land surface SM data and the in-situ measurement data from CERN indicated satisfactory performance of the ERA5-Land soil moisture product, with the mean Pearson correlation coefficients (R = 0.62). In Shandong Province, there is a CERN site: Yucheng Agroecosystem Station (YCA). Regional analysis at the YCA in Shandong Province revealed distinct seasonal performance: peak accuracy occurred in spring and autumn (R = 0.69), while summer and winter showed moderately reduced correlations (R = 0.55). (The definitions and computational methods for the R and RMSE are provided in the Methods section). Based on in-situ validation results, the ERA5-Land dataset demonstrates a strong capability in capturing the dynamic variations of soil moisture in Shandong Province. Its advantages in temporal consistency and spatial coverage make it well-suited for analyzing long-term spatiotemporal evolution patterns of soil moisture in this region.

In addition, the climate variables in the ERA5-Land database are generally in good agreement with ground-based observational data. In recent years, numerous scholars have verified the feasibility of precipitation, evaporation, and surface temperature sequences in the ERA5-Land reanalysis dataset [38–45]. Xu et al. [39] found through comparative evaluation that the precipitation estimates of ERA5 and ERA5-Land perform best in the temperate monsoon climate zone and the temperate continental climate zone on the Chinese mainland. For the ERA5-Land evapotranspiration data, Liu et al. [41] utilized eddy covariance data from 40 flux stations across different climatic regions in China to evaluate five reanalysis actual evapotranspiration datasets (ERA5, ERA5-Land, GLDAS-2.1, MERRA-2, Terra Climate). Their results showed that, geographically, ERA5-Land performed well in northern China but performed poorly in southern China. Moreover, Zou et al. [45] pointed out that the ERA5-Land temperature data effectively captures the daily, monthly, and interannual temperature variations across southeastern coastal China. Furthermore, among various land cover types, ERA5-Land demonstrates the best performance over farmland.

The Digital Elevation Model (DEM) data were obtained from the NASA Earthdata website. The NDVI data used in this study was obtained from the publicly available dataset published by [46], which can be accessed through the figshare platform. The ChinaEco100 Spatiotemporal Distribution Dataset of Ecosystem Types in China obtained from the Global Change Research Data Publishing & Repository, was used to assess the effects of ecosystem type on SM variation. The ecosystem classification includes farmland, forest, grassland, wetland, and settlement as shown in Fig 1. The soil classification for the study area was based on data from the Harmonized World Soil Database. Five soil groups (Luvisols, Cambisols, Regosols, Gleysols, and Solonchaks) accounted for 97% of the total area. All data processing and statistical analyses were performed using Python (version 3.11) with the aid of key scientific libraries, including pandas (version 2.2.2) for data cleaning and preprocessing, NumPy (version 1.26.4) for numerical computations, SciPy (version 1.13.1) for statistical analysis, and matplotlib (version 3.8.4) for data visualization.

### 2.3. Methods

#### 2.3.1. Generalized Additive Model (GAM).

The generalized additive model (GAM) is a statistical model that captures the complex relationship between independent variables and the response variable by introducing nonlinear smoothing functions. Relevant studies have shown that the GAM can be used to simulate the relationship between potential factors and SM. It can not only reflect the individual effect of each factor on SM but also assess the combined effect of various factors [4]. This study uses the GAM to evaluate these effects. The model can be expressed as follows:


$$\begin{array}{c} g\left({SM}_{i}\right)=\beta_{\text{0}}+s\left(P_{i}\right)+s\left(E_{i}\right)+s\left(T_{i}\right)+s\left({NDVI}_{i}\right)+\varepsilon \end{array}$$ (1)

Where, the ${SM}_{i}$ represents the soil moisture at grid i, $P_{i}$ is the precipitation (mm), $E_{i}$ is the evaporation (mm), $T_{i}$ is the surface temperature (°C), and the ${NDVI}_{i}$ represents the normalized difference vegetation index at grid i. The function s(x) denotes a smoothing function, ε is the residual error, and $\beta_{\text{0}}$ represents the overall mean response. The above data sequences were all monthly-scale data with the seasonal trend removed by using Seasonal-Trend decomposition based on Loess (STL) method. The GAM analysis was implemented using the statsmodels module (version 0.14.4) within the Python programming environment.

#### 2.3.2. Mann-Kendall test.

The Mann-Kendall (M-K) trend test is a non-parametric statistical test method, which is commonly used to quantify the significance of trends in hydrological and meteorological time series [47].Due to its characteristic that the sequence to be tested does not need to follow a specific distribution, it has been welcomed by many scholars. Previous studies have applied this method to trend analysis of SM [25]. For the detailed formula of the M-K trend test, please refer to the literature: [25]. After performing the M-K trend test on the sequence, the standard normal statistical variable (Z) is obtained. When the Z value is greater than 0, the sequence shows an increasing trend over time; when it is less than 0, the sequence shows a decreasing trend over time. At the 0.05 significance level (α = 0.05), when the absolute value of Z is greater than 1.96, it means that the sequence passes the 95% bilateral significance test. And the larger the absolute value of Z represents the more significant increasing or decreasing trend.

The Mann-Kendall abrupt change detection method is based on the monotonicity and volatility of the curves, and it can effectively identify abrupt change points in the time series and evaluate their significance. It is usually applied in the fields of environment, meteorology, hydrology, etc. [48,49]. For the detailed formula of the M-K abrupt change detection, please refer to the literature: [50].

Under the assumption of random independence in the time series, a statistical variable UF is defined, where UF > 0 indicates that the sequence shows an increasing trend and UF < 0 suggests a decreasing trend. By comparing UF with critical values (e.g., ± 1.96) corresponding to confidence level α = 0.05, if |UF| > 1.96, it indicates a significant change in trend. Subsequently, the inverse series statistic UB is calculated, where UB = -UF. If the intersection of the two curves is within the 95% confidence interval, it indicates that an abrupt change may occur at this time node. However, if multiple intersection points exist within the time interval without significant variation in the UF curve, they cannot be conclusively determined as abrupt change points. In this study, the Mann-Kendall trend test was performed on the data series using the pymannkendall package (version 1.4.3) within the Python programming environment.

#### 2.3.3. Correlation coefficient and cross-correlation coefficient.

Correlation analysis is a method used to measure the closeness of the relationship between two or more variables, which can explore the relationships among variables. The results are generally expressed in terms of correlation coefficients. For a sample $\left(X_{i},\;Y_{i}\right)$, the Pearson correlation coefficient is calculated using the following formula:


$$\begin{array}{c} r=\frac{\sum_{i=\text{1}}^{n}\left(X_{i}-\overset{―}{X}\right)\left(Y_{i}-\overset{―}{Y}\right)}{\sqrt{{\sum_{i=\text{1}}^{n}\left(X_{i}-\overset{―}{X}\right)}^{\text{2}}}\sqrt{{\sum_{i=\text{1}}^{n}\left(Y_{i}-\overset{―}{Y}\right)}^{\text{2}}}} \end{array}$$ (2)

In addition, by calculating the cross-correlation coefficient at different lag times, the lag time with the strongest correlation between the two time series can be identified, which can be used for lag analysis. For two time series X(t) and Y(t), the cross-correlation coefficient $R_{XY}(k)$ represents the correlation when Y(tis delayed by k time steps relative to X(t). The formula for calculating the cross-correlation coefficient is as follows:


$$\begin{array}{c} R_{XY}(k)=\frac{\sum_{t=\text{1}}^{N-K}\left(X(t)-\overset{―}{X}\right)\left(Y(t+k)\;-\;\overset{―}{Y}\right)}{\sqrt{\sum_{t=\text{1}}^{N}{\left(X(t)-\overset{―}{X}\right)}^{\text{2}}}\sqrt{\sum_{t=\text{1}}^{N}{\left(Y(t)-\overset{―}{Y}\right)}^{\text{2}}}} \end{array}$$ (3)

where X(t) and Y(t) are the two-time series. k is the lag time, representing the number of time steps by which Y(t) is delayed relative to X(t). N is the length of the time series. $\overset{―}{X}$ and $\overset{―}{Y}$ are the means of X(t) and Y(t), respectively. The analysis of correlation and cross-correlation coefficients was conducted employing the statsmodels module (version 0.14.4) within the Python programming environment.

#### 2.3.4. Wavelet analysis.

Wavelet analysis is a widely used time-frequency analysis tool, which can be used to analyze periodic characteristics [51]. The wavelet transform overcomes the limitations of the Fourier transform in time-frequency localization and is particularly suitable for processing non-stationary signals [52,53]. At present, wavelet analysis has been widely used in image processing, seismic signal analysis [54], hydrological modeling [55], and other fields.

The real part of wavelet coefficients usually contains the main features of the signal, so the contour map of the real part of wavelet coefficients can intuitively observe the periodic variations at a certain time scale. The curve in the wavelet variance plot reflects the distribution of the periodic fluctuation energy of the sequence with the time scale. Using continuous wavelet transform (CWT), we can obtain wavelet coefficients and plot the contour map of the real part of wavelet coefficients and the map of wavelet variance. The mathematical definition is as follows:


$$\begin{array}{c} W(a\;,\;b)=\;\int_{-\infty }^{\infty }f(t)\frac{\text{1}}{\sqrt{a}}\phi \left(\frac{t-b}{a}\right)dt \end{array}$$ (4)

Where W(a , b) is the wavelet coefficient. a is the scale parameter. b is the translation parameter. f(t) is the signal (time series) to be analyzed. The function ϕ(x) is a basic wavelet function. In this study, the complex Morlet wavelet selected, which combines a cosine wave and a Gaussian function, is suitable for periodicity analysis. The wavelet analysis for each sequence was conducted utilizing the PyWavelets package (version 1.8.0) within the Python environment.

## 3. Results

### 3.1. Influence of climatic and environmental variables on SM variation

In this section, this study first assessed the effects of climatic variables (precipitation, evaporation, and temperature) and environmental factors (landform, ecosystem type, soil category, and vegetation index) on SM variation. Then, the entire study area was partitioned according to qualitative variables (landform, ecosystems, and soil types), and the GAM was used to explore the individual and combined effects of quantitative variables (precipitation, evaporation, temperature, and NDVI) on SM variation.

By comparing the correlation coefficients between elevation and monthly soil moisture, we found that the correlation between surface soil moisture and altitude was greater during the spring-winter season (from December to May) (with an average R value of 0.34) than that during the summer-autumn season (from June to November) (with an average R value of 0.28). This reflected that altitude is also to some extent correlated with the surface soil moisture in Shandong Province. As shown in the research of Guo et al. [56], soil moisture is also affected by altitude and land use. Therefore, we divided Shandong Province into flatland and mountainous regions according to different altitudes.

**Fig 2:**
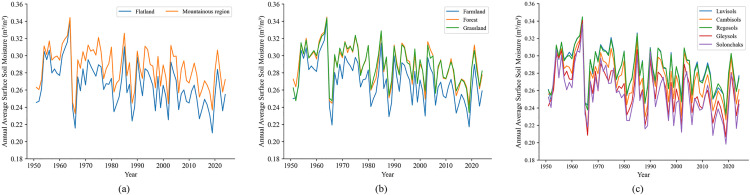
Interannual variations of surface soil moisture across different (a) landforms, (b) ecosystems, and (c) soil types.

Fig 2 illustrate that the inter-annual variations of surface soil moisture in different landforms, different ecosystems, and different soil types have the same changing trend. It can also be seen that the surface soil moisture content in the flatland (almost all farmland) is relatively low. In addition, the results of the M-K trend test indicate that the soil moisture in different classified regions shows a significant decreasing trend.

**Fig 3:**
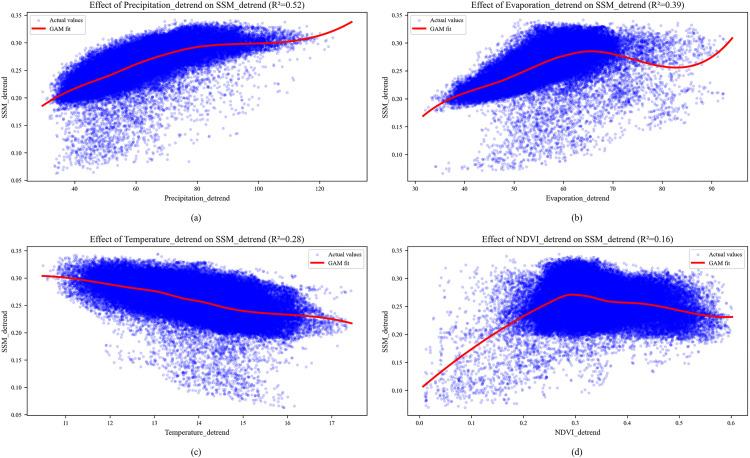
Individual effects on surface soil moisture variability (p = 0.000). **(a)** precipitation, **(b)** evaporation, **(c)** temperature, **(d)** NDVI.

Using monthly-scale data from 1982 to 2022, this study employed GAM to quantify the individual and combined contributions of climate (precipitation, evaporation, temperature) and vegetation (NDVI) to soil moisture variation across Shandong Province, alongside the influences of landform, ecosystem, and soil type. All time-series variables were de-seasoned to isolate underlying trends, enabling robust analysis of parameter variations and enhanced detection of synergistic effects among drivers. Both the individual effects of each factor and the combined effects of all factors were measured by the coefficient of determination R^2^, and the significance of each factor was determined by the p-value of the likelihood ratio statistic. The individual effect of SM spatiotemporal variability was shown in Fig 3. At monthly scale, precipitation (R^2^ = 0.52) exhibited the strongest explanatory power, indicating that precipitation processes accounted for 52% of soil moisture variability in the study area, making it the dominant controlling factor. The evaporation (R^2^ = 0.39) and temperature (R^2^ = 0.28) were secondary factors. The NDVI (R^2^ = 0.16) had a relatively weak explanatory capacity.

Based on the individual effect of each dominant driving factor on the spatiotemporal variability of SM, we further examine their combined effects at a monthly scale across the study region. The combined effects of precipitation, evaporation, temperature, and NDVI explained 72% of the spatiotemporal variability in SM within the study region, significantly outperforming any individual factor’s explanatory power.

A comparative evaluation was conducted to examine how these four factors, both independently and collectively, influence SM dynamics in different landforms (Flatlands, Mountains), major ecosystem types (Farmland, Forest and Grassland), and soil types (Luvisols, Cambisols, Regosols, Gleysols, and Solonchaks). The results from the GAM implementation are presented in Table 1.

**Table 1:** Explanatory power (R^2^) of driving factors. Individual versus combined effects on soil moisture variability across landforms (Flatlands, Mountains), ecosystems (Farmland, Forest, Grassland), and soil types (Luvisols, Cambisols, Regosols, Gleysols, and Solonchaks).

Region	Temperature (T)	Evaporation (E)	Precipitation (P)	NDVI	Combined factors
Whole study region	0.28	0.39	0.52	0.16	0.72
Flatlands	0.2	0.37	0.5	0.16	0.68
Mountainous	0.19	0.35	0.57	0.09	0.68
Farmland	0.29	0.45	0.57	0.12	0.73
Forest	0.2	0.32	0.61	0.14	0.7
Grassland	0.17	0.14	0.41	0.31	0.71
Luvisols	0.16	0.26	0.45	0.07	0.57
Cambisols	0.24	0.53	0.65	0.06	0.8
Regosols	0.25	0.31	0.47	0.15	0.66
Gleysols	0.23	0.55	0.64	0.09	0.77
Solonchaks	0.24	0.14	0.17	0.36	0.68

As shown in Table 1, the explanatory patterns remained consistent across most subdivisions (excluding grassland ecosystems and Solonchaks soil group), with the individual and combined effects of P, E, T, and NDVI on SM spatiotemporal variability showing little change. Among these, climate factors exerted the strongest driving force on SM within Cambisols soil group. In grassland ecosystems, although precipitation (P, 41%) remained the dominant driver of SM dynamics, the explanatory power of NDVI (31%) increased notably, while the influences of evaporation (E, 14%) and temperature (T, 17%) weakened. Compared to farmland and forest ecosystems, grassland ecosystems may exhibit a tighter coupling between vegetation cover and soil moisture. This relationship can form a positive feedback loop: soil moisture drives plant growth and increases vegetation cover, which in turn improves the micro-environment by modulating evaporation and infiltration, ultimately enhancing soil water retention. In Solonchaks soil group, the NDVI was the dominant driver of SM dynamics. Sparse vegetation cover strongly exacerbates water evaporation and salt accumulation, while any recovery of vegetation can markedly suppress this process through shading. Therefore, compared to other soil groups, even minor changes in vegetation cover can more directly drive changes in soil moisture by exerting a strong regulatory control over the evaporation process. However, given the limited areal coverage of both grassland ecosystems and the solonchaks soil group, variations specific to these ecosystems and soil types did not significantly alter the explanatory power of the overall drivers at the regional scale. This suggests that the observed P-E-T-NDVI hierarchy is robust across the entire study area. Based on the individual and combined effects analysis of driving factors on SM spatiotemporal variability, the comparatively weak explanatory power of NDVI prompted us to focus specifically on the spatiotemporal patterns and correlations between SM and the three climatic factors.

### 3.2. Temporal distribution and correlation analysis of SM and climatic variables

#### 3.2.1. Interannual variation of the annual average SM and climatic variables.

**Fig 4:**
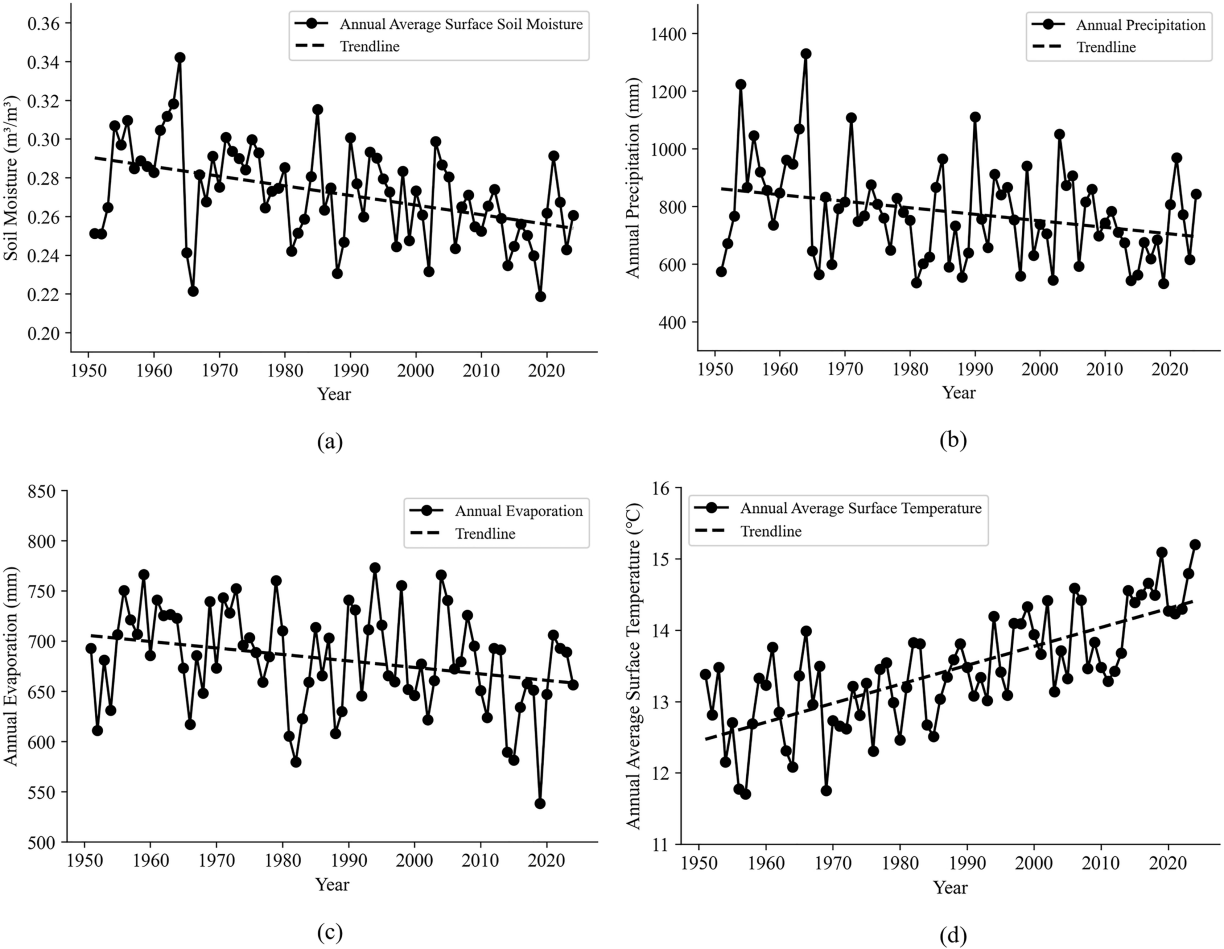
Interannual variation trends of annual (a) average surface soil moisture, (b) precipitation, (c) evaporation and (d) average surface temperature.

Firstly, this study calculated the annual average surface soil moisture content, annual precipitation, annual evaporation, and annual average surface temperature series for the whole Shandong Province from 1951 to 2024 and visualized as Fig 4. Fig 4 reveals distinct temporal trends among the analyzed variables: In Fig 4(A), the annual mean surface soil moisture exhibits a declining trend with interannual fluctuations, suggesting increasing soil aridity over the study period. In Fig 4(B) and Fig 4(C), both annual precipitation and evaporation display substantial interannual variability, yet maintain overall decreasing trends. In contrast, Fig 4(D) shows that the annual mean surface temperature demonstrates a pronounced warming trend, indicating accelerated regional warming.

Subsequently, to improve the science and precision of the SM and climatic factors’ trend characteristic analysis, this study further employed the M-K trend test to analyze the interannual variation trends of four sequences (the annual average surface soil moisture content, annual precipitation, annual evaporation, and annual average surface temperature) in Shandong Province from 1951 to 2024. The results of the M-K trend test were shown in Table 2.

**Table 2:** Results of the Mann-Kendall trend test.

Sequence (Annual)	Statistical quantity (Z)	Trend (1951-2024)	Significance Level (p)
Surface Soil Moisture	−3.9761	Decrease	0.0001
Precipitation	−2.3240	Decrease	0.0201
Evaporation	−2.2867	Decrease	0.0222
Surface Temperature	6.5708	Increase	0.0000

According to the results of the M-K trend test, the trend changes of the four sequences are consistent with the visualization results. For the annual precipitation and annual evaporation sequences, the significance p-values are less than 0.05, passing the significance test at the confidence level (α) of 0.05. For the annual average surface soil moisture content and the annual average surface air temperature sequences, the significance p-values are less than 0.001, passing the significance test at a 99.9% confidence level, indicating that the changes in these two sequences are more significant. Therefore, for the entire Shandong Province, between 1951 and 2024, the decreasing rate of the annual average surface soil moisture content was −0.00055 mm/year, the decreasing rate of the annual precipitation was −2.1339 mm/year, the decreasing rate of the annual evaporation was −0.6464 mm/year, and the increasing rate of the annual average surface temperature was 0.02747°C/year. All sequences showed significant trend changes.

**Fig 5:**
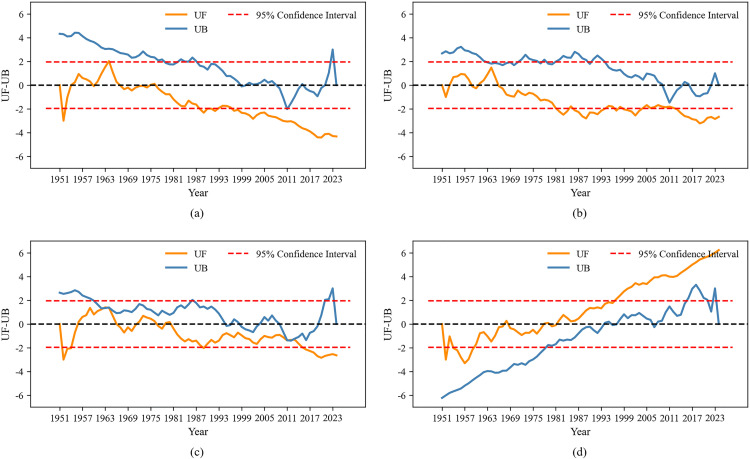
The UF-UB curves of annual (a) average surface soil moisture, (b) precipitation, (c) evaporation and (d) average surface temperature.

Next, to further examine the significance and abrupt change points of the four sequences within a certain year interval, this study employed the Mann-Kendall abrupt change analysis method for detection. The results of the M-K abrupt change analysis were shown in Table 3 and Fig 5.

**Table 3:** Results of the Mann-Kendall abrupt change detection.

Sequence (Annual)	Location of the UF-UB Intersection
Surface Soil Moisture	None
Precipitation	None
Evaporation	1962, 2012
Surface Temperature	None

Combining Table 3 and Fig 5, we can observe the following trends for the annual average surface soil moisture (Fig 5(A)). From 1951 to 1977, soil moisture showed a fluctuating trend of both decline and increase. After 1978, the UF values remain negative and exceed the critical value of −1.96 in 1988, indicating a significant decreasing trend in the annual average surface soil moisture content. However, the UF and UB curves did not intersect within the 95% significance interval, suggesting that there was no significant abrupt change point in the annual average surface soil moisture content.

For the annual precipitation (Fig 5(B)), between 1951 and 1966, the UF value was negative first and then positive, showing a trend of first decreasing and then increasing. After 1966, the UF values remain below 0 and exceeded the critical value of −1.96 in 1982, indicating a significant decrease in annual precipitation during this period. Similar to the annual average surface soil moisture, the UF and UB curves did not intersect within the 95% significance interval, and thus no significant abrupt change point was detected.

In Fig 5(C), the UF and UB curves of annual evaporation intersect within the 95% confidence interval in 1962 and 2012, respectively. Moreover, the UF curve exceeds the critical value of −1.96 in 2014, indicating that significant abrupt changes in annual evaporation occurred in 1962 and 2012.

Fig 5(D) reflected that the UF-UB curves for the annual average surface temperature do not intersect, indicating that there are no significant abrupt changes years during 1951–2024. From 1951 to 1981, the UF values of the annual average surface temperature are generally less than or equal to 0, suggesting a decreasing trend during this period. After 1982, the UF values are all greater than 0, and the critical value of 1.96 was exceeded in 1997, indicating a significant increasing trend in surface temperature from 1982 to 2024.

#### 3.2.2. Interannual variation among seasons of SM.

**Fig 6:**
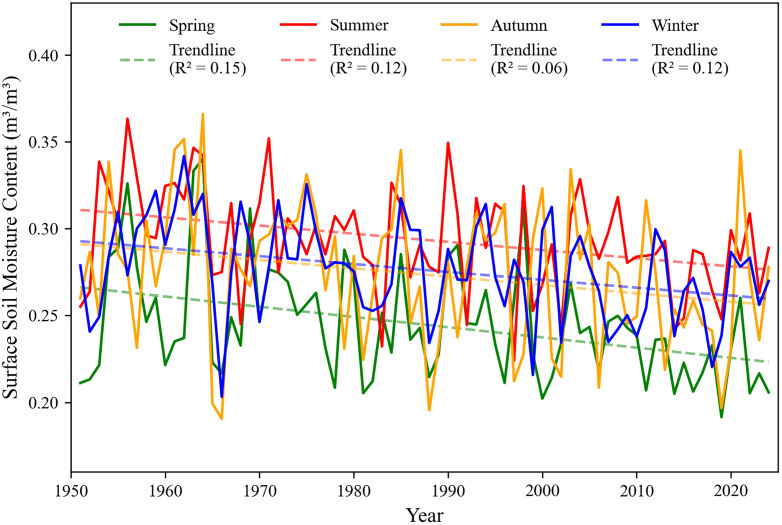
Trend of seasonal variation of surface soil moisture content. Spring (March, April, May); Summer (June, July, August); Autumn (September, October, November); Winter (December, January, February).

This study conducted a time series analysis of the surface soil moisture content in Shandong Province from 1951 to 2024, which revealed a strong seasonal periodicity by removing the interannual variation trend of the surface soil moisture time series. Therefore, this study divided the data of the surface soil moisture content from January 1951 to December 2024 into four seasons according to the months: spring (March, April, May), summer (June, July, August), autumn (September, October, November), and winter (December, January, February), and calculated the surface soil moisture content for each season of each year. As shown in Fig 6, the interannual variation trends of the average surface soil moisture content in different seasons in Shandong Province from 1951 to 2024.

It can be seen from Fig 6 that the surface soil moisture content in different seasons shows a decreasing trend with year. Moreover, the surface soil moisture is the highest in summer, while the surface soil moisture in spring is obviously lower than that in the other three seasons. Since spring is the critical period for local crop sowing, the seasonal variation of surface soil water reflects the influence of agricultural irrigation and crop growth. The soil moisture content in autumn and winter is similar, with that in winter being slightly higher, probably due to the supplementation of soil moisture from snowfall caused by cold weather.

To further scientifically study the decreasing trends and rates of each season over the years, this study used the M-K trend test to analyze the trends of the surface soil moisture content series for each season. The results of the M-K trend test were shown in Table 4.

**Table 4:** Results of the M-K trend test for SM in different seasons.

Seasons	Statistical quantity (Z)	Trend (1951–2024)	Significance Level (p)
Spring	−3.2387	Decrease	0.0012
Summer	−2.8654	Decrease	0.0042
Autumn	−2.1560	Decrease	0.0311
Winter	−2.9680	Decrease	0.0030

It can be seen from Table 4 that the statistical Z-values of the average surface soil moisture sequences in the four seasons are all less than 0, and the significance p-values are all less than 0.05, indicating that they pass the significance test at the confidence level of 0.05, showing a significant decreasing trend. So based on the M-K trend test, during the period from 1951 to 2024, the average surface soil moisture in spring decreased significantly at a rate of −0.00054 mm/year; the significant decreasing rate in summer was −0.00049 mm/year; the significant decreasing rate in autumn was −0.00051 mm/year; and in winter, it decreased significantly at a rate of −0.00048 mm/year. Among them, the significance p-values for the sequences of surface soil moisture content in spring, summer, and winter were all less than 0.01, indicating that the decrease in these three seasons was more significant compared to autumn.

#### 3.2.3. Temporal dynamics of the relationships between SM and climatic factors.

**Fig 7:**
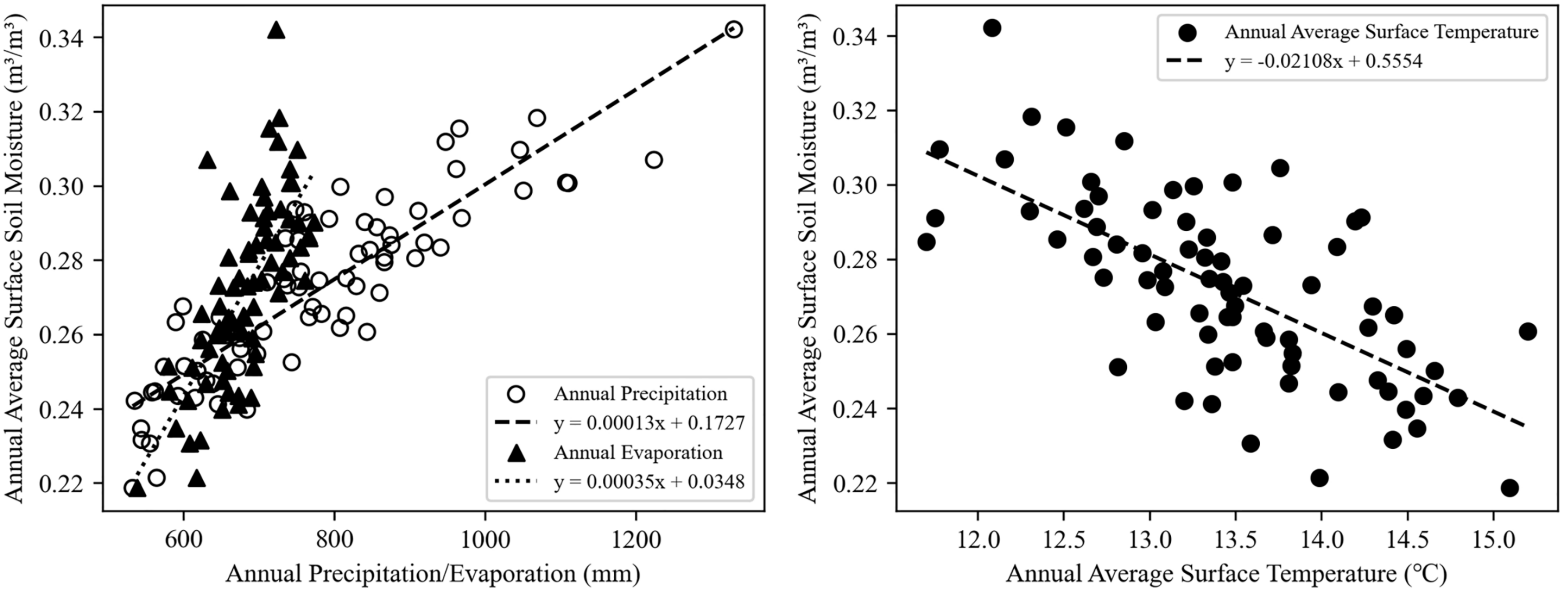
The scatter plot between the annual average surface soil moisture and three climatic factors.

Correlation analysis can quickly clarify the degree of influence of various driving factors on soil moisture. Fig 7 illustrates the relationship between the annual average surface soil moisture content and annual precipitation, annual evaporation, and annual average surface temperature from 1951 to 2024. It can be clearly observed that the soil moisture content is directly proportional to both precipitation and evaporation. Specifically, as precipitation increased, the soil moisture content rose, and evaporation also increased. In addition, a clear inverse relationship was evident between soil moisture content and surface temperature.

To further quantitatively investigate the correlation between soil moisture and the three factors, this study calculated the correlation coefficients for analysis. The results and tests of the correlation coefficients were shown in Table 5.

**Table 5:** Pearson correlation coefficients between SM and three climatic factors.

Sequence	Correlation Coefficient	Significance Level (p)
SM and P	0.879^**^	0.000
SM and E	0.710^**^	0.000
SM and T	−0.675^**^	0.000

** The correlation is significant at the 0.01 level.

According to the calculation results of the correlation coefficients in Table 5, it can be found that the correlation coefficient between soil moisture content and precipitation is 0.879 (p < 0.01); the correlation coefficient with evaporation is 0.710 (p < 0.01); and the correlation coefficient with surface temperature is −0.675 (p < 0.01). All of these have passed the 99% significance test, indicating that there are significant strong correlations between surface soil moisture content and three factors. Among them, the correlation coefficient between surface soil moisture and precipitation is the largest, showing an extremely strong correlation.

### 3.3. Spatial distribution and correlation analysis of SM and climatic variables

#### 3.3.1. Spatial distribution of SM and climatic variables.

**Fig 8:**
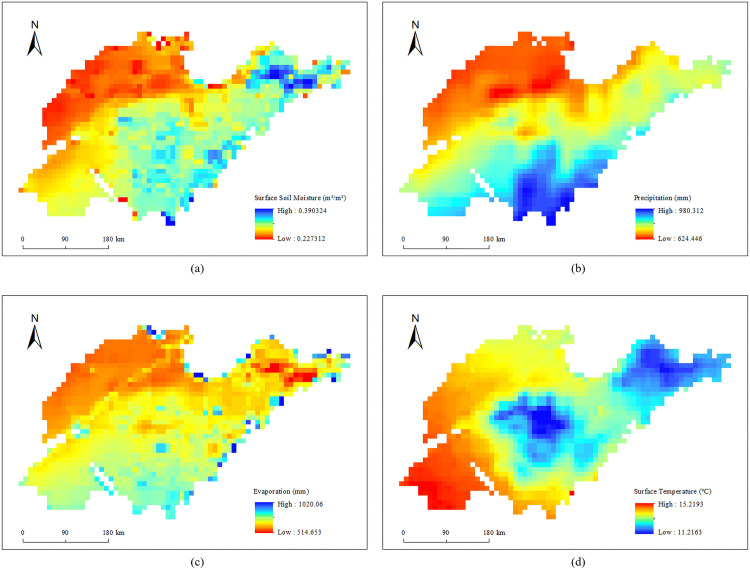
Spatial distribution of multi-year average (a) surface soil moisture, (b) precipitation, (c) evaporation, and (d) surface temperature.

To further analyze the spatial distribution of soil moisture content in Shandong Province, this study calculated the multi-year average surface soil moisture content, multi-year average precipitation, multi-year average evaporation, and multi-year average surface temperature for each grid from 1951 to 2024, and visualized the results as shown in Fig 8.

The average surface soil moisture content in Shandong Province from 1951 to 2024 generally decreased from the southeast coast to the northwest inland, which showed a strong consistency with the spatial distribution of the multi-year average annual precipitation and evaporation. Combined with the DEM data of Shandong Province, it was found that the spatial distribution of the annual average surface temperature was relatively consistent with the landform distribution. The central and northeastern regions have higher elevations, resulting in lower annual average surface temperatures, where the surface soil moisture content is relatively higher. In contrast, the northwest and southwest regions are predominantly plains and closer to the inland areas, leading to higher surface temperatures and lower surface soil moisture content.

#### 3.3.2. Spatial distribution of correlation coefficient between SM and climatic variables.

**Fig 9:**
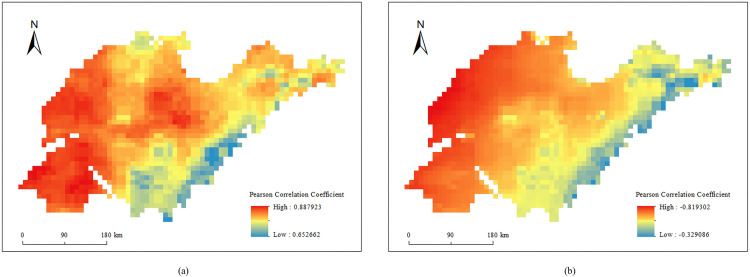
Spatial distribution of the Pearson correlation coefficient between surface soil moisture and (a) precipitation, (b) surface temperature.

Due to the spatial heterogeneity of the large-scale research objects [57], the impact of climatic factors on surface soil moisture are also reflected in their spatial distribution. Thus, for the research object of this study—Shandong Province, it is necessary to explore the spatial distribution of the correlation coefficients. Accordingly, this study investigated the spatial distribution of the correlation coefficients between surface soil moisture and annual precipitation and annual average surface temperature, as shown in Fig 9.

The result indicates that in the western region of Shandong Province, surface soil moisture is strongly positively correlated with precipitation and strongly negatively correlated with surface temperature, while the surface soil moisture in the coastal areas is relatively less influenced by these two factors.

**Fig 10:**
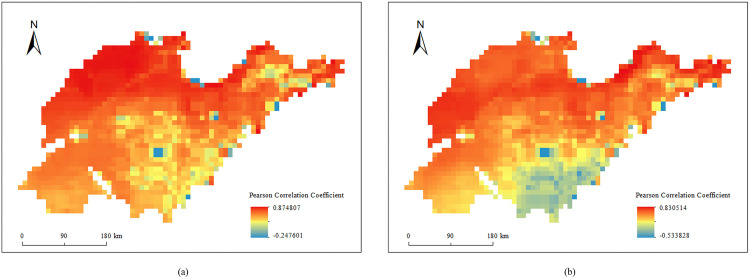
Spatial distribution of the Pearson correlation coefficient between surface soil moisture and evaporation. **(a)** dry season, **(b)** wet season.

However, the impact of evaporation on soil moisture content is more complex in space, showing both positive and negative correlations. This may be related to the coupling effect between evaporation and precipitation. Therefore, this study divided the seasons into the dry season (Spring) and the wet season (Summer) based on the level of soil moisture content and calculated the correlation coefficients with the corresponding evaporation amounts. The spatial distribution maps of the correlation coefficients for the dry and wet seasons were presented in Fig 10. The results indicate that in the northwestern part of Shandong Province, the surface soil moisture shows a very strong positive correlation with evaporation during both the dry and wet seasons (correlation coefficient > 0.8). In contrast, in the southeastern part of Shandong Province, the soil moisture content exhibits a positive correlation with evaporation in the dry season but a negative correlation in the wet season.

### 3.4. Periodic analysis of SM and climatic variables

**Fig 11:**
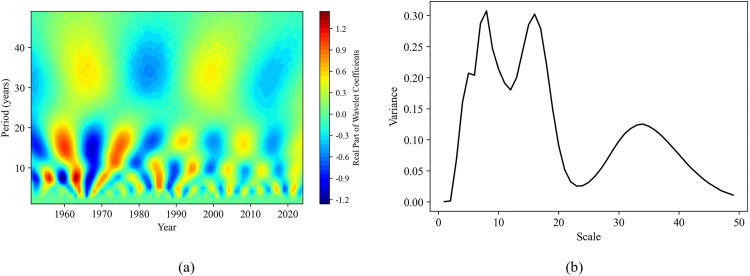
Wavelet analysis of surface soil moisture content sequence. **(a)** The real part isolines map of wavelet coefficients; **(b)** Wavelet coefficient variance map.

**Fig 12:**
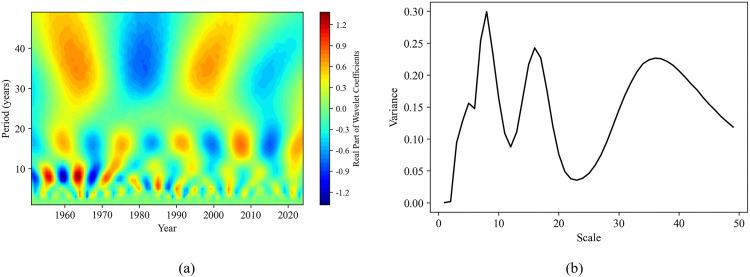
Wavelet analysis of annual precipitation sequence. **(a)** The real part isolines map of wavelet coefficients; **(b)** Wavelet coefficient variance map.

**Fig 13:**
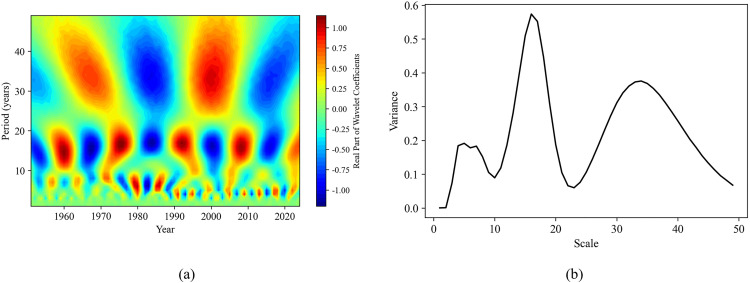
Wavelet analysis of annual evaporation sequence. **(a)** The real part isolines map of wavelet coefficients; **(b)** Wavelet coefficient variance map.

**Fig 14:**
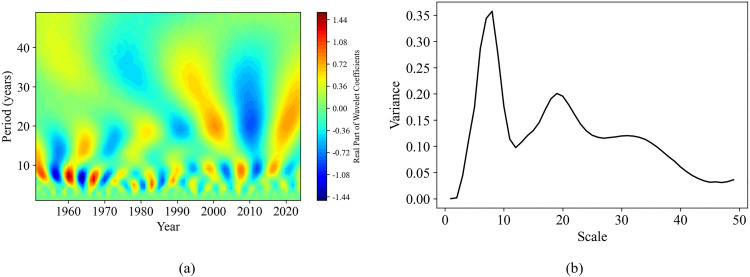
Wavelet analysis of annual average surface temperature sequence. **(a)** The real part isolines map of wavelet coefficients; **(b)** Wavelet coefficient variance map.

Combining the contour map of the real part of the wavelet coefficients and the wavelet coefficient variance map can help analyze the periodic variation patterns of sequences at different time scales. This is of great significance for understanding the dynamic changes in soil moisture content and the study of related environmental factors. In this study, the continuous wavelet transform (complex Morlet wavelet) was used to obtain the contour map of wavelet coefficient real parts and the wavelet coefficient variance map for each sequence (Figs 11-14).

In Fig 11(B), there are three obvious maxima on the wavelet coefficient variance map of the surface soil moisture content sequence, indicating the presence of three dominant periods (time scales): 8 years, 16 years, and 33 years. Among them, the wavelet coefficient variances of 6 years and 16 years are both the largest, which are considered the first dominant period; the 33 years is regarded as the second dominant period. Combined with Fig 11(A), it can be more clearly observed that at the time scale of 8 years, there is a period of about 7–8 years from 1951 to 1970 and from 1982 to 1990. At the time scale of 16 years, there is an approximately 16 years period from 1951 to 1985, while after 1990, the 16-year period becomes relatively less pronounced. At the time scale of 33 years, a period of about 32–33 years exists for the entire period, and the fainter colors in the contour map indicate weaker energy variations in this time domain, making the cycle less obvious.

Fig 12(B) reflects the wavelet coefficient variance map of the annual precipitation sequence, which also has three peaks, indicating the existence of dominant periods (time scales) of 8 years, 16 years, and 37 years. Among them, the first dominant period is 8 years, and the second 16 years and 37 years. By combining Fig 12(A), it can be observed that, at the time scale of 8 years, there exists a period of about 8 years from 1951 to 1973, while the period variation becomes relatively less obvious after 1974. At the 16 years and 37 years scales, periods of about 16 years and 36–37 years exist respectively throughout the entire period.

There are three different peaks at 4–7 years, 16 years and 33 years in the wavelet coefficient variance map of Fig 13(B), indicating that the annual evapotranspiration sequence has three dominant periods. Of these, the first dominant period is 16 years, the second is 33 years, and the third is 4–7 years. From Fig 13(A) it can be observed that, at the time scale of 16 years, there exists a period of about 15–16 years from 1951 to 2024; at the time scale of 33 years, there is a period of 31–33 years for the whole period; at the time scale of 7 years, there exists a period of 5–6 years from 1975 to 1990; and 4 years period approximately during 1991–1998 and 2005–2024.

For Fig 14(B), there are only two peaks in the wavelet coefficient variance map, 8 years (the first dominant period) and 19 years (the second dominant period). We combined these two-time scales with the contour map of the real parts of wavelet coefficients (Fig 14(A)), and found that for the annual average surface temperature sequence there exists an about 7–8 years period during 1951–1971, 1980–1990, and 2000–2020 at the time scale of 8 years; there exists an approximately 16–19 years period throughout the entire time period at the time scale of 19 years.

### 3.5. Time-lagged effects of climatic factors on SM dynamics

Two time series may contain hidden correlation information, and the characterization of these correlations is important for understanding their nature and mechanisms [58]. The causal relationship between soil moisture content, precipitation, evaporation, and surface temperature can be determined by time-lagged correlation. By calculating the cross-correlation coefficients with positive and negative lags between them, we can more comprehensively understand the dynamic relationship between the two-time series This not only helps analyze the response mechanisms of soil moisture to climatic factors across different time scales but also provides a theoretical foundation for precise irrigation and agricultural drought warning. Therefore, this study conducted a time-lag cross-correlation analysis on the annual and monthly soil moisture content and climatic factor sequences from 1951 to 2024.

**Fig 15:**
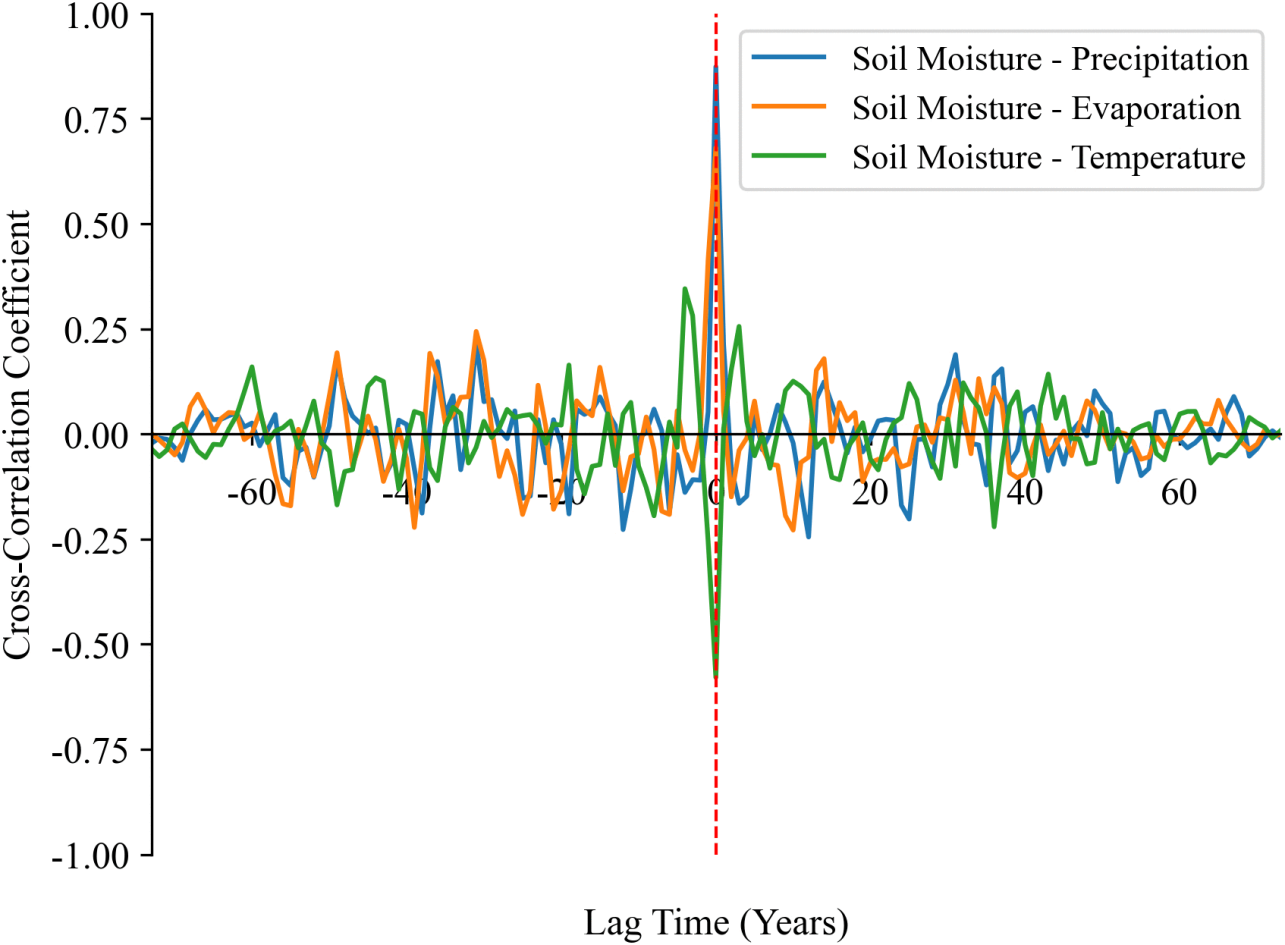
Cross-correlation coefficients between soil moisture and annual precipitation, evaporation, and average surface temperature.

Fig 15 shows the variation of cross-correlation coefficients between the surface soil moisture sequence and the annual precipitation, annual evaporation, and annual average surface temperature sequences with the lag time. In Fig 15, the cross-correlation of surface soil moisture with both annual precipitation and evaporation peaks at zero lag, revealing an immediate response at the annual scale. The cross-correlation of surface soil moisture with both precipitation and evaporation peaks at zero lag, revealing an immediate response, while the strongest negative correlation with temperature also occurs without lag, indicating a synchronous inverse relationship. Moreover, this study further explored the responses of the annual-scale sequences of soil moisture in the lower and deep layers to these three climatic factors. The results all showed immediate correlations.

**Fig 16:**
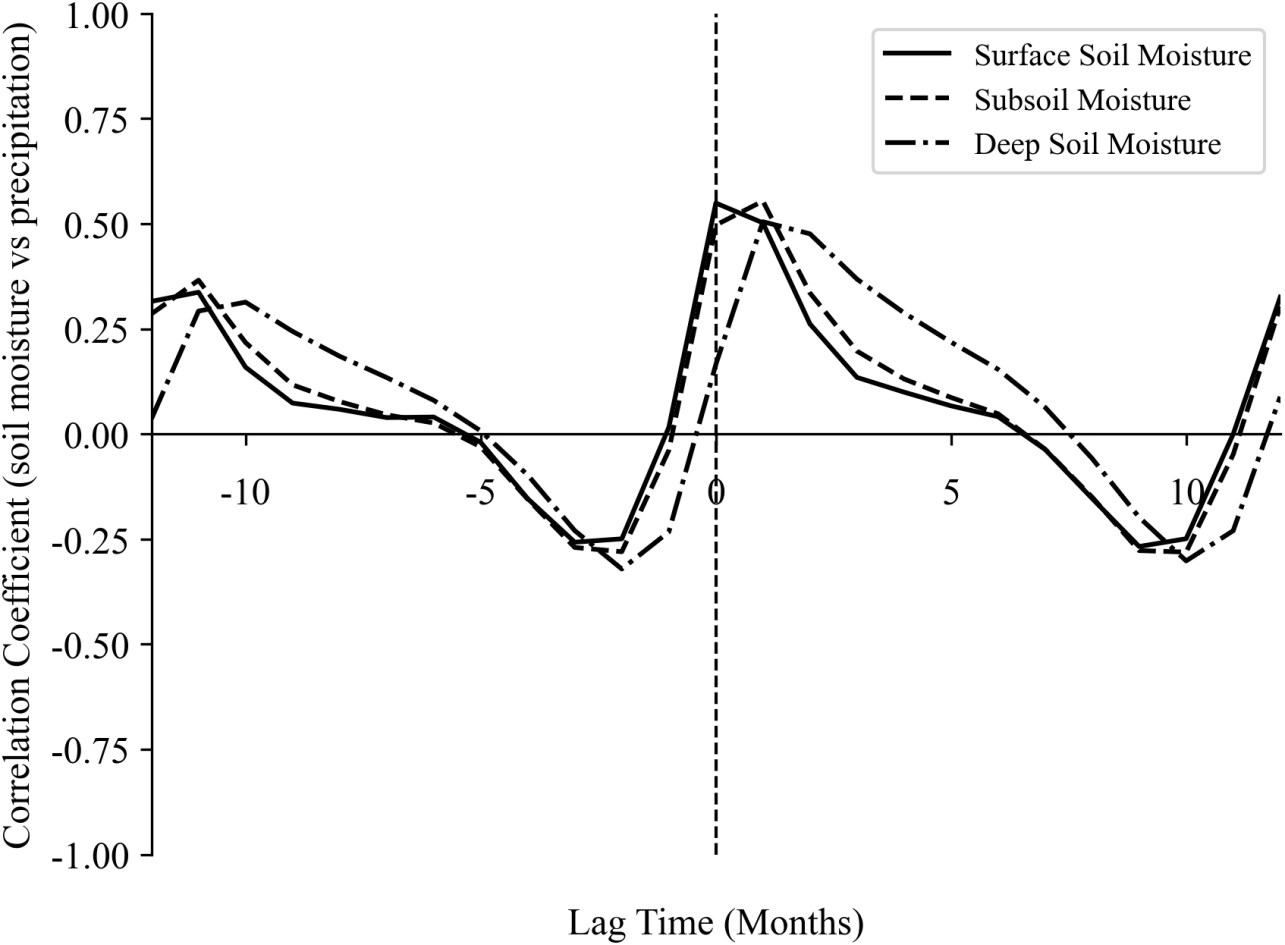
Lag response of soil moisture at different depths to precipitation at the monthly scale.

Combined with the above analysis, this study took into account the size of the time scale and the effect of soil moisture content at different depths, so it further explored the correlations on the monthly lagged correlation between the three-layer soil moisture content sequence and the corresponding monthly cumulative precipitation sequence (Fig 16). The results showed that, on a monthly scale the surface soil moisture (0–7 cm) lags 0 month behind precipitation, the subsoil moisture (7–28 cm) lags by 0–1 month relative to precipitation, and the deep layer soil moisture (28–100 cm) lags by 1–2 months relative to precipitation. This indicates that soil moisture at different depths has different lag times on a monthly scale, with deeper soil moisture exhibiting a more pronounced lag effect in response to precipitation.

**Fig 17:**
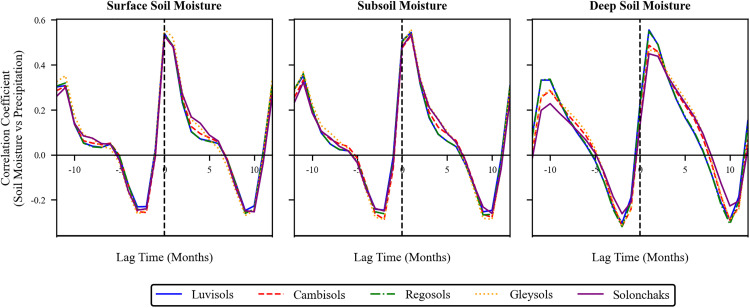
Monthly lag response of soil moisture to precipitation across soil depths and types.

Furthermore, to address the potential confounding effect of soil heterogeneity in time-lagged correlation analysis, this study specifically investigated the SM response to precipitation across five soil groups (Luvisols, Cambisols, Regosols, Gleysols, and Solonchaks). The results indicated an immediate SM-precipitation correlation across all soil layers on an annual scale. On a monthly scale, the lag time of SM to precipitation increased with soil depth (Fig 17). Notably, despite this general pattern, the overarching lag trend was consistent across all five soil types and aligned with the regional-scale pattern. This suggests that soil type has a limited influence on the lagged response dynamics in the study area.

## 4. Discussion

This study assessed the effects of climatic and environmental factors on SM variations, revealing that climatic variables were the dominant controls of spatiotemporal SM variability in Shandong Province, China. Consequently, this study provided an in-depth examination of the spatiotemporal patterns and interrelationships between soil moisture and key climatic factors (temperature, precipitation and evapotranspiration), while highlighting their critical implications for regional water resource management and agricultural practices.

On a monthly scale, the correlation between soil moisture and altitude showed seasonal variations. The average correlation coefficients were 0.34 in winter-spring and 0.28 in summer-autumn, respectively (both statistically significant, p < 0.01). This suggests that lower temperatures may reduce evaporation, potentially enhancing the regulatory effect of altitude on soil water retention. Overall, soil moisture was positively correlated with altitude, consistent with the findings of Li et al. [33]. Higher-altitude regions generally experience cooler temperatures and increased precipitation (e.g., orographic rainfall on windward slopes), leading to greater soil moisture. Combining Fig 1 and Fig 2(A-B), it was found that the surface soil moisture content in the flatland (almost all farmland) is relatively low, which indirectly reflects that agriculture in Shandong Province has a relatively high demand for water resources. Fig 2(B) shows that the average soil moisture content of grassland is the highest, which is consistent with the research results of Guo et al. [56]. However, their study reported forests as the lowest, while farmland exhibited the lowest values in our case. This revealed that there were certain differences in the impact of land use in different research areas on soil moisture. These discrepancies may stem from divergent climatic conditions, soil properties, topography (e.g., slope gradient), and land management practices (e.g., irrigation in farmland). The climatic and edaphic conditions in Shandong Province (e.g., lower precipitation, loess-dominated soils) contrast sharply with the Northeast China Black Soil Region (e.g., higher humidity, fertile mollisols), likely explaining the divergent results.

To further quantify the drivers of soil moisture, we applied Generalized Additive Models both across the entire study area and separately for distinct ecosystem types and soil groups. The results show that both ecosystem-specific and soil group-variations similarly fail to significantly alter the overall explanatory power of the driving factors at the regional scale. Moreover, based on the analysis of the individual effects of various driving factors on the spatiotemporal variability of soil moisture, the results indicate that precipitation is the most significant driver of soil moisture (R^2^ = 0.52, p < 0.01), consistent with hydrological cycle theory in monsoonal climates like Shandong Province, where seasonal rainfall strongly replenishes soil moisture. The secondary roles of evaporation (R^2^ = 0.39, p < 0.01) and temperature (R^2^ = 0.28, p < 0.01) align with energy-limited evapotranspiration regimes, mediated by thermal and radiative controls [59]. NDVI’s explanatory power is weakest in farmland (R^2^ = 0.12, p < 0.01), likely due to irrigation decoupling vegetation-soil moisture linkages, whereas its effect is significantly higher in natural Grasslands (R^2^ = 0.31, p < 0.01). Distinctly different from the driving pattern observed in other subdivisions, NDVI is identified as the dominant driver for soil moisture dynamics in the Solonchaks soil group. Compared to other ecosystem and soil types, the regulatory effect of vegetation cover on soil moisture is markedly stronger in Grasslands and Solonchaks. Despite this localized influence, our comparison of individual and combined driving factors (Table 1) reveals that climatic factors are the primary drivers of soil moisture variability. This dominance holds across different altitudes, soil groups and ecosystems.

The average surface soil moisture in Shandong Province exhibited a fluctuating decreasing trend over the years. This observation aligns with findings from related studies conducted in other regions of China, such as the North China region [28], the Huang-Huai-Hai Plain [22], and the karst region [60]. Furthermore, it also aligned with the research indicating an overall decreasing trend in global soil moisture [4,5]. In addition to the inter-annual trends, the surface soil moisture demonstrated distinct seasonal characteristics, with the highest SM in summer and the lowest in spring, which verified the seasonal characteristics of soil moisture in northern China as studied by Yao et al. [22]. Notably, the finding that soil moisture reached its lowest level in spring and showed a significant decreasing trend over the years holds particular importance for agricultural planning. This is because spring is a crucial sowing period in Shandong Province. The relatively low soil moisture content during this period may exacerbate the risk of drought [61]. Therefore, it is necessary to adopt adaptive irrigation strategies in spring to ensure crop yields. Moreover, as shown in Table 5, precipitation exerts the most significant influence on surface soil moisture. This result was consistent with studies conducted in semi-arid regions such as Xinjiang [26] and the Loess Plateau [60]. It also verified the conclusion drawn by Li et al. (2025) on a global scale: in mid-latitude and low-latitude regions, soil moisture content is primarily influenced by precipitation [4].

Cho et al. (2014) emphasized that the spatial variability of soil moisture is significantly influenced by meteorological factors [3]. Shandong Province is located within the monsoon region of China. The southeast coastal region is affected by the monsoon, which brings a large amount of precipitation, thus increasing the soil moisture content. Additionally, the uneven spatial distribution of the correlation coefficient between soil moisture and meteorological factors highlights the influence of topography and local microclimates: SM in coastal areas was less affected by precipitation and surface temperature, whereas SM correlates closely with these two factors. This conclusion was supported by the research of Cho and Choi [3]. Notably, the relationship between surface soil moisture and evaporation in the southeast coastal region was complex. Specifically, soil moisture was positively correlated with evaporation in the dry season, but negatively correlated in the rainy season. This may be related to the coupling effect of evaporation and precipitation [62].

Based on the results of wavelet transform (Figs 11-14), it is revealed that the periodic pattern of soil moisture at the time scale of 8 years was consistent with the dominant oscillations of precipitation and surface temperature. So, soil moisture was obviously influenced by these two factors over an 8 years scale. This short-term periodic variation may be related to the extreme climate change caused by the El Niño-Southern Oscillation (ENSO) phenomenon, which has a quasi-periodicity of 2–7 years. On the other hand, the periodicity at the 16 years scale was the same as that of annual evaporation, indicating that surface soil moisture was also affected by evaporation over longer time scales. These periodic change patterns can predict how soil moisture will change in the next few years, providing a certain basis and reference for drought monitoring in the study area. In addition, by comparing the contour map of the real parts of the wavelet coefficients of the evaporation sequence with that of the precipitation sequence, we found that there was a complementary relationship between the strong and weak variations in the periodicity of the annual evaporation and the annual precipitation. Overall, the periodicities (8 years, 16 years, and 32 years) identified in soil moisture variations were consistent with the dominant oscillations of precipitation and evaporation. This consistency indicates that climate changes driven by phenomena such as El Niño and La Niña may be the key factors causing short-term variations in soil moisture [4].

After conducting a lag analysis, we found that there was an immediate correlation between soil moisture and the three climatic factors on an annual scale. Then, by comparing the maximum cross-correlation coefficients between soil moisture and the three climatic factors, we found that the maximum cross-correlation coefficient between the soil moisture sequence and the precipitation sequence was higher than that with the other two sequences. This further indicates that the response of soil moisture to precipitation is the most significant. Additionally, on a monthly scale, soil moisture at different depths responds differently to precipitation. The deeper the soil moisture, the more significant the lag effect in response to precipitation. Among them, the deep soil (28–100 cm) has a lagged response to precipitation of 1–2 months, which means it can buffer short-term drought to a certain extent. In agricultural production, the water storage capacity of deep soil can be enhanced through deep plowing and water-conservation technologies (such as straw mulching) to reduce the impact of seasonal drought on crop roots. Some scholars have conducted further research on this lag phenomenon. For example, Zhu et al. [63] investigated the response of different soil depths to precipitation in the gully region of the Loess Plateau and found that the lag time is mainly influenced by precipitation amount, precipitation intensity, and initial soil water content. Therefore, the importance of soil depth also needs to be considered when assessing drought risk or irrigation needs. Furthermore, human activities, particularly artificial water diversion for irrigation, can alter the lagged response of soil moisture to precipitation. However, the timing of irrigation varies significantly across different agricultural zones and crop types. This heterogeneity introduces considerable complexity, necessitating further dedicated research to isolate and quantify the impact of anthropogenic irrigation on the soil moisture dynamics in cultivated regions.

This study elucidates the long-term spatiotemporal patterns of soil moisture and their relationships with key driving factors in Shandong Province, offering scientific insights for agricultural production and drought monitoring. Nonetheless, its scope is constrained by a focus on climatic and environmental factors (e.g., altitude, landform, ecosystem type, soil type, and vegetation), a provincial-scale study area, and annual/monthly time scales. Consequently, future research should expand the geographical scope from a single province to broader regions and employ data with finer spatial and temporal resolutions, while also incorporating key anthropogenic drivers like irrigation, to better characterize both regional patterns and localized soil moisture dynamics.

## 5. Conclusions

In this study, the spatiotemporal patterns of soil moisture in Shandong Province have been explored using ERA5-Land reanalysis data. The conclusions are obtained as follows:

(1) Precipitation was the main controlling factor in Shandong Province, evaporation and temperature were secondary factors, and the explanatory power of the NDVI was relatively weak. The annual mean surface soil moisture showed a significant decreasing trend with year, and no significant abrupt change points were detected. In different seasons, surface soil moisture showed a significant decreasing trend with year and it was lowest in spring. Moreover, the surface SM content in flatland (almost all farmland) was relatively low.
(2) Spatially, the surface soil moisture in Shandong Province generally decreased from the southeast coast to the northwest, aligning with the spatial distributions of the multi-year average annual precipitation and evaporation. In the west, the surface soil moisture was greatly affected by precipitation and surface temperature, while in the coastal area, these factors had less impact. Additionally, it correlated positively with evaporation during dry seasons but negatively during wet seasons in the southeast.
(3) The CWT analysis revealed that soil moisture exhibited a period of about 8 years, 16 years, and 32 years. Combined with the CWT of precipitation, evaporation, and surface temperature, it was found that soil moisture was significantly influenced by precipitation and surface temperature at the time scale of 8a, while it was also affected by evaporation at the time scale of 16a.
(4) The cross-correlation analysis of the time lag showed that there was an immediate correlation between soil moisture and precipitation, evaporation, and surface temperature on the annual scale. Further investigation into the monthly scale response of soil moisture to precipitation revealed that the deeper the soil layer, the more pronounced the lag in response to precipitation.

Overall, the findings of this study—including the long-term spatiotemporal trends and periodic characteristics of soil moisture—can enhance agricultural productivity and water-use efficiency in Shandong Province. These insights can inform adjustments to crop planting and irrigation practices, such as implementing water-saving irrigation in spring. They also provide a scientific basis for water resource management, supporting both short-term measures like strategic water storage and long-term projects like the Yellow River to Qingdao Diversion. Ultimately, this work contributes to achieving China’s agricultural water-saving goals for 2030.

## References

[1] Liu Q, Wang T, Liu C, Mikouendanandi EMRB, Chen X, Peng T, et al. Characterizing the spatiotemporal dynamics of shallow soil water stable isotopic compositions on a karst hillslope in Southwestern China. Journal of Hydrology. 2022;610:127964. doi: 10.1016/j.jhydrol.2022.127964

[2] Brocca L, Melone F, Moramarco T, Morbidelli R. Spatial‐temporal variability of soil moisture and its estimation across scales. Water Resources Research. 2010;46(2). doi: 10.1029/2009wr008016

[3] Cho E, Choi M. Regional scale spatio-temporal variability of soil moisture and its relationship with meteorological factors over the Korean peninsula. Journal of Hydrology. 2014;516:317–29. doi: 10.1016/j.jhydrol.2013.12.053

[4] Li Y-X, Leng P, Kasim AA, Li Z-L. Spatiotemporal variability and dominant driving factors of satellite observed global soil moisture from 2001 to 2020. Journal of Hydrology. 2025;654:132848. doi: 10.1016/j.jhydrol.2025.132848

[5] Peng C, Zeng J, Chen K-S, Li Z, Ma H, Zhang X, et al. Global spatiotemporal trend of satellite-based soil moisture and its influencing factors in the early 21st century. Remote Sensing of Environment. 2023;291:113569. doi: 10.1016/j.rse.2023.113569

[6] Elbialy S, Mahmoud A, Pradhan B, Buchroithner M. Application of spaceborne synthetic aperture radar data for extraction of soil moisture and its use in hydrological modelling at Gottleuba Catchment, Saxony, Germany. J Flood Risk Management. 2013;7(2):159–75. doi: 10.1111/jfr3.12037

[7] Zhang K, Zhao L, Yang K, Qin J, Song L, Ni X, et al. Spatiotemporal scales of precipitation in the Central Tibetan Plateau identified by in-situ soil moisture observations. Journal of Hydrology. 2023;626:130319. doi: 10.1016/j.jhydrol.2023.130319

[8] Zhang K, Wang Q, Chao L, Ye J, Li Z, Yu Z, et al. Ground observation-based analysis of soil moisture spatiotemporal variability across a humid to semi-humid transitional zone in China. Journal of Hydrology. 2019;574:903–14. doi: 10.1016/j.jhydrol.2019.04.087

[9] Holgate CM, de Jeu RAM, van Dijk AIJM, Liu YY, Renzullo LJ, Vinod K, et al. Comparison of Remotely Sensed and Modelled Soil Moisture Data Sets across Australia. Remote Sensing Environ. 2016;186, 479–500.doi: 10.1016/j.rse.2016.09.015

[10] Gu X, Li J, Chen YD, Kong D, Liu J. Consistency and Discrepancy of Global Surface Soil Moisture Changes From Multiple Model‐Based Data Sets Against Satellite Observations. JGR Atmospheres. 2019;124(3):1474–95. doi: 10.1029/2018jd029304

[11] Edokossi K, Calabia A, Jin S, Molina I. GNSS-Reflectometry and Remote Sensing of Soil Moisture: A Review of Measurement Techniques, Methods, and Applications. Remote Sensing. 2020;12(4):614. doi: 10.3390/rs12040614

[12] Ochsner TE, Cosh MH, Cuenca RH, Dorigo WA, Draper CS, Hagimoto Y, et al. State of the Art in Large-Scale Soil Moisture Monitoring. Soil Science Society of America Journal. 2013;77(6):1888–919. doi: 10.2136/sssaj2013.03.0093

[13] Zhang R, Li L, Zhang Y, Huang F, Li J, Liu W, et al. Assessment of Agricultural Drought Using Soil Water Deficit Index Based on ERA5-Land Soil Moisture Data in Four Southern Provinces of China. Agriculture. 2021;11(5):411. doi: 10.3390/agriculture11050411

[14] Kim H, Wigneron J-P, Kumar S, Dong J, Wagner W, Cosh MH, et al. Global scale error assessments of soil moisture estimates from microwave-based active and passive satellites and land surface models over forest and mixed irrigated/dryland agriculture regions. Remote Sensing of Environment. 2020;251:112052. doi: 10.1016/j.rse.2020.112052

[15] Wang Y, Zhao H, Fan J, Wang C, Ji X, Jin D, et al. A Review of Earth’s Surface Soil Moisture Retrieval Models via Remote Sensing. Water. 2023;15(21):3757. doi: 10.3390/w15213757

[16] Albergel C, de Rosnay P, Gruhier C, Muñoz-Sabater J, Hasenauer S, Isaksen L, et al. Evaluation of remotely sensed and modelled soil moisture products using global ground-based in situ observations. Remote Sensing of Environment. 2012;118:215–26. doi: 10.1016/j.rse.2011.11.017

[17] Kim H, Parinussa R, Konings AG, Wagner W, Cosh MH, Lakshmi V, et al. Global-scale assessment and combination of SMAP with ASCAT (active) and AMSR2 (passive) soil moisture products. Remote Sensing of Environment. 2018;204:260–75. doi: 10.1016/j.rse.2017.10.026

[18] Beck HE, Pan M, Miralles DG, Reichle RH, Dorigo WA, Hahn S, et al. Evaluation of 18 satellite- and model-based soil moisture products using in situ measurements from 826 sensors. Hydrol Earth Syst Sci. 2021;25(1):17–40. doi: 10.5194/hess-25-17-2021

[19] Muñoz-Sabater J, Dutra E, Agustí-Panareda A, Albergel C, Arduini G, Balsamo G, et al. ERA5-Land: a state-of-the-art global reanalysis dataset for land applications. Earth Syst Sci Data. 2021;13(9):4349–83. doi: 10.5194/essd-13-4349-2021

[20] Wu Z, Feng H, He H, Zhou J, Zhang Y. Evaluation of Soil Moisture Climatology and Anomaly Components Derived From ERA5-Land and GLDAS-2.1 in China. Water Resour Manage. 2021;35(2):629–43. doi: 10.1007/s11269-020-02743-w

[21] Zhou X, Hu K, Xiao H, Yang Y, Chen J, Cheng Y. Effects of vegetation on the spatiotemporal distribution of soil water content in re-vegetated slopes using temporal stability analysis. CATENA. 2024;234:107570. doi: 10.1016/j.catena.2023.107570

[22] Yao XL, Jiang Q, Liu Y, Li LY, Wang QY, Yu JS. Spatiotemporal variation of soil moisture in Northern China based on climate change initiative data. Agronomy Journal. 2021;113(2):774–85. doi: 10.1002/agj2.20479

[23] Wang Y, Cao J, Liu Y, Zhu Y, Fang X, Huang Q, et al. Spatiotemporal Analysis of Soil Moisture Variation in the Jiangsu Water Supply Area of the South-to-North Water Diversion Using ESA CCI Data. Remote Sensing. 2022;14(2):256. doi: 10.3390/rs14020256

[24] Wang L, Lu J, Zhou R, Duan G, Wen Z. Analysis of Soil Moisture Change Characteristics and Influencing Factors of Grassland on the Tibetan Plateau. Remote Sensing. 2023;15(2):298. doi: 10.3390/rs15020298

[25] Geng M, Zhang F, Chang X, Wu Q, Liang L. Spatial-temporal Variation of Soil Moisture in China from Long Time Series Based on GLDAS-Noah. Sensors and Materials. 2021;33(12):4643. doi: 10.18494/sam.2021.3445

[26] Hu Z, Chen X, Li Y, Zhou Q, Yin G. Temporal and Spatial Variations of Soil Moisture Over Xinjiang Based on Multiple GLDAS Datasets. Front Earth Sci. 2021;9. doi: 10.3389/feart.2021.654848

[27] Kumar M, Sahu AP, Paul JC, Dash SS, Sahoo BC, Nayak AK, et al. Assessment of Long-term spatiotemporal soil moisture variation in the lower Mahanadi River basin: a hydrological modeling based approach. Environ Dev Sustain. 2024. doi: 10.1007/s10668-024-05030-5

[28] Cai J, Zhou B, Chen S, Wang X, Yang S, Cheng Z, et al. Spatial and Temporal Variability of Soil Moisture and Its Driving Factors in the Northern Agricultural Regions of China. Water. 2024;16(4):556. doi: 10.3390/w16040556

[29] Hu Y, Tang C, Chen X, Zhao Y, He H, Li M, et al. Spatiotemporal Variability of Soil Water Content and Its Influencing Factors on a Microscale Slope. Agronomy. 2023;13(8):2035. doi: 10.3390/agronomy13082035

[30] Liu B, Shao M. Response of soil water dynamics to precipitation years under different vegetation types on the northern Loess Plateau, China. J Arid Land. 2015;8(1):47–59. doi: 10.1007/s40333-015-0088-y

[31] Wei X, Gao J, Liu S, Zhou Q. Temporal Variation of Soil Moisture and Its Influencing Factors in Karst Areas of Southwest China from 1982 to 2015. Water. 2022;14(14):2185. doi: 10.3390/w14142185

[32] Su B, Wang A, Wang G, Wang Y, Jiang T. Spatiotemporal variations of soil moisture in the Tarim River basin, China. International Journal of Applied Earth Observation and Geoinformation. 2016;48:122–30. doi: 10.1016/j.jag.2015.06.012

[33] Li L, Wu D, Wang T, Wang Y. Effect of topography on spatiotemporal patterns of soil moisture in a mountainous region of Northwest China. Geoderma Regional. 2022;28:e00456. doi: 10.1016/j.geodrs.2021.e00456

[34] Wang Q, Liu Y, Zhu G, Lu S, Chen L, Jiao Y, et al. Regional differences in the effects of atmospheric moisture residence time on precipitation isotopes over Eurasia. Atmospheric Research. 2025;314:107813. doi: 10.1016/j.atmosres.2024.107813

[35] Chen L, Zhu G, Lin X, Li R, Lu S, Jiao Y, et al. The Complexity of Moisture Sources Affects the Altitude Effect of Stable Isotopes of Precipitation in Inland Mountainous Regions. Water Resources Research. 2024;60(6). doi: 10.1029/2023wr036084

[36] Xing Z, Li X, Fan L, Colliander A, Frappart F, De Rosnay P, et al. Assessment of 9 km SMAP soil moisture: Evidence of narrowing the gap between satellite retrievals and model-based reanalysis. Remote Sensing of Environment, 2023;296, 113721. doi: 10.1016/j.rse.2023.113721

[37] Fan L, Xing Z, Lannoy GD, Frappart F, Peng J, Zeng J, et al. Evaluation of satellite and reanalysis estimates of surface and root-zone soil moisture in croplands of Jiangsu Province, China. Remote Sensing of Environment. 2022;282:113283. doi: 10.1016/j.rse.2022.113283

[38] Wang D, Wang D, Liu S, Huang Y, Yang Q, Ma X, et al. Comprehensive evaluation of nine evapotranspiration products from remote sensing, gauge upscaling and land surface model over China. PLoS One. 2024;19(11):e0313762. doi: 10.1371/journal.pone.0313762 39536035 PMC11560003

[39] Xu J, Ma Z, Yan S, Peng J. Do ERA5 and ERA5-land precipitation estimates outperform satellite-based precipitation products? A comprehensive comparison between state-of-the-art model-based and satellite-based precipitation products over mainland China. Journal of Hydrology. 2022;605:127353. doi: 10.1016/j.jhydrol.2021.127353

[40] Chang Y, Qi Y, Wang Z. Comprehensive evaluation of IMERG, ERA5-Land and their fusion products in the hydrological simulation of three karst catchments in Southwest China. Journal of Hydrology: Regional Studies. 2024;52:101671. doi: 10.1016/j.ejrh.2024.101671

[41] Liu Y, Wang W, Zhao T, Huo Z. Performance Evaluation and Spatiotemporal Dynamics of Nine Reanalysis and Remote Sensing Evapotranspiration Products in China. Remote Sensing. 2025;17(11):1881. doi: 10.3390/rs17111881

[42] Tan ML, Armanuos AM, Ahmadianfar I, Demir V, Heddam S, Al-Areeq AM, et al. Evaluation of NASA POWER and ERA5-Land for estimating tropical precipitation and temperature extremes. Journal of Hydrology. 2023;624:129940. doi: 10.1016/j.jhydrol.2023.129940

[43] Wang Y-R, Hessen DO, Samset BH, Stordal F. Evaluating global and regional land warming trends in the past decades with both MODIS and ERA5-Land land surface temperature data. Remote Sensing of Environment. 2022;280:113181. doi: 10.1016/j.rse.2022.113181

[44] Yilmaz M. Accuracy assessment of temperature trends from ERA5 and ERA5-Land. Sci Total Environ. 2023;856(Pt 2):159182. doi: 10.1016/j.scitotenv.2022.159182 36195144

[45] Zou J, Lu N, Jiang H, Qin J, Yao L, Xin Y, et al. Performance of air temperature from ERA5-Land reanalysis in coastal urban agglomeration of Southeast China. Sci Total Environ. 2022;828:154459. doi: 10.1016/j.scitotenv.2022.154459 35278562

[46] Li H, Cao Y, Xiao J, Yuan Z, Hao Z, Bai X, et al. A daily gap-free normalized difference vegetation index dataset from 1981 to 2023 in China. Sci Data. 2024;11(1):527. doi: 10.1038/s41597-024-03364-3 38778028 PMC11111700

[47] Gocic M, Trajkovic S. Analysis of changes in meteorological variables using Mann-Kendall and Sen’s slope estimator statistical tests in Serbia. Global and Planetary Change. 2013;100:172–82. doi: 10.1016/j.gloplacha.2012.10.014

[48] Liu Z, Yang H, Wei X, Liang Z. Spatiotemporal Variation in Extreme Precipitation in Beijiang River Basin, Southern Coastal China, from 1959 to 2018. JMSE. 2023;11(1):73. doi: 10.3390/jmse11010073

[49] Wang J. Determining the most accurate program for the Mann-Kendall method in detecting climate mutation. Theor Appl Climatol. 2020;142(3–4):847–54. doi: 10.1007/s00704-020-03333-x

[50] Fattahi E, Kamali S, Asadi Oskouei E, Habibi M. Investigating the Spatiotemporal Variation in Extreme Precipitation Indices in Iran from 1990 to 2020. Water. 2025;17(8):1227. doi: 10.3390/w17081227

[51] Meng Y, Sun W. Relationship between the Formation of PM2.5 and Meteorological Factors in Northern China: The Periodic Characteristics of Wavelet Analysis. Advances in Meteorology. 2021;2021:1–14. doi: 10.1155/2021/9723676

[52] Guo T, Zhang T, Lim E, Lopez-Benitez M, Ma F, Yu L. A Review of Wavelet Analysis and Its Applications: Challenges and Opportunities. IEEE Access. 2022;10:58869–903. doi: 10.1109/access.2022.3179517

[53] Li B, Chen X. Wavelet-based numerical analysis: A review and classification. Finite Elements in Analysis and Design. 2014;81:14–31. doi: 10.1016/j.finel.2013.11.001

[54] Zheng W, Li X, Yin L, Yin Z, Yang B, Liu S, et al. Wavelet analysis of the temporal-spatial distribution in the Eurasia seismic belt. Int J Wavelets Multiresolut Inf Process. 2017;15(03):1750018. doi: 10.1142/s0219691317500187

[55] Shoaib M, Shamseldin AY, Khan S, Sultan M, Ahmad F, Sultan T, et al. Input Selection of Wavelet-Coupled Neural Network Models for Rainfall-Runoff Modelling. Water Resour Manage. 2018;33(3):955–73. doi: 10.1007/s11269-018-2151-x

[56] Guo X, Fu Q, Hang Y, Lu H, Gao F, Si J. Spatial Variability of Soil Moisture in Relation to Land Use Types and Topographic Features on Hillslopes in the Black Soil (Mollisols) Area of Northeast China. Sustainability. 2020;12(9):3552. doi: 10.3390/su12093552

[57] Luo P, Song Y, Huang X, Ma H, Liu J, Yao Y, et al. Identifying determinants of spatio-temporal disparities in soil moisture of the Northern Hemisphere using a geographically optimal zones-based heterogeneity model. ISPRS Journal of Photogrammetry and Remote Sensing. 2022;185:111–28. doi: 10.1016/j.isprsjprs.2022.01.009

[58] Shen C. Analysis of detrended time-lagged cross-correlation between two nonstationary time series. Physics Letters A. 2015;379(7):680–7. doi: 10.1016/j.physleta.2014.12.036

[59] Seneviratne SI, Corti T, Davin EL, Hirschi M, Jaeger EB, Lehner I, et al. Investigating soil moisture–climate interactions in a changing climate: A review. Earth-Science Reviews. 2010;99(3–4):125–61. doi: 10.1016/j.earscirev.2010.02.004

[60] Wang X, Wang B, Xu X, Liu T, Duan Y, Zhao Y. Spatial and temporal variations in surface soil moisture and vegetation cover in the Loess Plateau from 2000 to 2015. Ecological Indicators. 2018;95:320–30. doi: 10.1016/j.ecolind.2018.07.058

[61] Whitmore AP, Whalley WR. Physical effects of soil drying on roots and crop growth. J Exp Bot. 2009;60(10):2845–57. doi: 10.1093/jxb/erp200 19584120

[62] Oueslati B, Bony S, Risi C, Dufresne J-L. Interpreting the inter-model spread in regional precipitation projections in the tropics: role of surface evaporation and cloud radiative effects. Clim Dyn. 2016;47(9–10):2801–15. doi: 10.1007/s00382-016-2998-6

[63] Zhu P, Zhang G, Wang H, Zhang B, Liu Y. Soil moisture variations in response to precipitation properties and plant communities on steep gully slope on the Loess Plateau. Agricultural Water Management. 2021;256:107086. doi: 10.1016/j.agwat.2021.107086

